# Genome Sequencing of Kocuria varians Strain 80, Isolated from Starter Culture for Dry Sausage

**DOI:** 10.1128/MRA.00086-21

**Published:** 2021-02-25

**Authors:** Evgenii A. Konorov, Konstantin A. Kurbakov, Mikhail Y. Minaev

**Affiliations:** a V. M. Gorbatov Federal Research Center for Food Systems, Russian Academy of Sciences, Moscow, Russia; b Vavilov Institute of General Genetics, Russian Academy of Sciences, Moscow, Russia; University of Delaware

## Abstract

The genome of the meat starter culture strain Kocuria varians 80 was sequenced and assembled into a single 2.82-Mb chromosome. Genome sequence comparison of *K. varians* strain 80 and previously sequenced strains revealed predicted proteomic differences that can impact its technological properties.

## ANNOUNCEMENT

Kocuria varians is used as a starter culture for processed meat and dairy products. In combination with other starter cultures or by itself, it can participate in proteolysis, denitrification, and biogenic amine degradation (1–3), which leads to changes in the flavor and aroma of a processed product. Recent genome studies of starter cultures have succeeded in describing the biotechnological properties of starter cultures. Among them are the meat starter culture strains Staphylococcus carnosus TM300 (4), Staphylococcus xylosus (5), Lactobacillus sakei (6), and others. Here, we present the genomic characteristics of Kocuria varians strain 80 (SSCA-2165).

Kocuria varians strain 80 was received from State Scientific Center for Antibiotics Culture (SSCA; culture number 2165) and was originally isolated from dried fermented sausages in 1978 (7). The strain was grown in 1 ml of Trypticase soy broth (TSB) (Liofilchem, Roseto degli Abruzzi, Italy) at 30°C for 24 h. The culture was inoculated onto mannitol salt agar (MSA; Liofilchem) and incubated at 30°C for 48 h. Then, one colony was inoculated in a tube with 5 ml TSB and incubated at 30°C for 48 h.

Bacterial DNA was isolated with the GBD kit (Biosilica, Russia). We used the NEBNext Ultra DNA library prep kit (New England Biolabs, USA) for Illumina HiSeq 2500 sequencing. For PacBio RS II single-molecule real-time (SMRT) sequencing, we used the SMRTbell template prep kit v1.0 (Pacific Biosciences, USA) following the 20-kb template preparation protocol using the BluePippin size selection system. In total, there were 2.1 billion (2 × 250-bp) Illumina reads and 158,304 PacBio reads (average read length, 7.6 kb).

After read and adapter trimming with Trimmomatic v0.36 (8) with the command line parameters ILLUMINACLIP:TruSeq3-PE.fa:2:30:10 LEADING:3 TRAILING:3 SLIDINGWINDOW:4:20 MINLEN:100, the genome sequence of *K. varians* was assembled with SPAdes v3.11 (9), with error correction using both PacBio and Illumina reads. Gene annotation was conducted with NCBI Prokaryotic Genome Annotation Pipeline v4.12 (10); every predicted gene product was subjected to a BLASTp v2.9.0 (11) search against the NCBI nonredundant (nr) database and an InterProScan v5.33-72.0 (12) search. Gene Ontology and KEGG pathway annotations were performed using Blast2GO v4 (13) for *K. varians* strain 80, *K*. *salsicia* G1 (GenBank accession number GCA_001408335.1), and *K. varians* G6 (GCA_900014985.1). After circularization using Simple Circularise v1.0 (https://github.com/Kzra/Simple-Circularise), the genome assembly consisted of a single contig with a 2,823,038-bp circular chromosome (assembly coverage, 47.9×). The genome has a 70.55% GC content. We found 2,465 protein-coding sequences, 48 tRNA genes, and 9 rRNA genes.

Unlike *K. varians* C6, both *K. varians* 80 and *K. salsicia* G1 contain the *xylB* gene, according to KEGG pathway analysis. Also unlike *K. varians* G6 and *K. salsicia* G1, strain 80 has a complete metabolic pathway from d-glucose-1P to l-rhamnose, which is potentially important to its properties as a meat-fermenting starter culture.

### Data availability.

The PacBio and Illumina reads were submitted to the SRA under accession numbers SRR13177364 and SRR13177365, respectively. The genome sequence of *K. varians* strain 80 (SSCA-2165) has been deposited at GenBank under accession number GCF_002250745.2.
